# Development and internal validation of a macrophage migration inhibitory factor-based risk assessment model for delayed cerebral ischemia in patients with aneurysmal subarachnoid hemorrhage

**DOI:** 10.3389/fsurg.2026.1922721

**Published:** 2026-09-15

**Authors:** JianBo Zhang, ChengMing Bao, AnKui Lu, Kun Guo

**Affiliations:** 1 Department of Neurosurgery, Nanjing Lishui People’s Hospital, Nanjing, Jiangsu, China; 2 Department of Ultrasound Medicine, Nanjing Lishui People’s Hospital, Nanjing, Jiangsu, China; 3 Department of Medical Imaging, Nanjing Lishui People’s Hospital, Nanjing, Jiangsu, China

**Keywords:** aneurysmal subarachnoid hemorrhage, delayed cerebral ischemia, left ventricular ejection fraction, macrophage migration inhibitory factor, mFisher grade, nomogram, risk assessment model

## Abstract

**Background:**

Delayed cerebral ischemia (DCI) is an important cause of poor outcomes in patients with aneurysmal subarachnoid hemorrhage (aSAH). Macrophage migration inhibitory factor (MIF) is involved in the regulation of inflammatory responses and may be associated with the development of DCI after aSAH. This study aimed to investigate the association between admission serum MIF levels and DCI in patients with aSAH and to develop an MIF-based risk assessment model for DCI.

**Methods:**

Patients with aSAH were included and divided into DCI and non-DCI groups according to the occurrence of DCI during hospitalization. Demographic characteristics, aneurysm-related features, hemorrhage burden indicators, admission laboratory parameters, and cardiac function parameters were collected. Baseline characteristics were compared between the two groups, and logistic regression analyses were performed to identify factors associated with DCI. A nomogram was constructed based on the final multivariable model, and model discrimination, calibration, and clinical utility were evaluated.

**Results:**

Among 100 patients with aSAH, 28 developed DCI. The DCI group had a higher proportion of patients with high modified Fisher grades, higher admission MIF levels, and lower LVEF. Several factors were associated with increased DCI risk, including elevated MIF, high modified Fisher grade, reduced LVEF, intraventricular hemorrhage, acute hydrocephalus, elevated white blood cell count, and elevated hs-cTnI. In the final model, elevated MIF, high modified Fisher grade, and reduced LVEF remained independently associated with DCI. The three-variable model demonstrated an AUC higher than that of any single predictor, acceptable calibration after bootstrap internal validation, and potential net benefit across clinically relevant thresholds.

**Conclusion:**

Increased admission MIF, high modified Fisher grade, and decreased LVEF were independently associated with higher DCI risk in patients with aSAH. An internally validated model incorporating these three indicators may offer a simple, interpretable tool for early DCI risk stratification. Its generalizability and clinical applicability require confirmation in larger, multicenter prospective cohorts.

## Introduction

In neurocritical care, aneurysmal subarachnoid hemorrhage (aSAH) represents a serious cerebrovascular event characterized by abrupt onset, severe clinical manifestations, numerous complications, and high risks of both disability and death (1). Global epidemiological investigations have revealed geographic variations in aSAH incidence, and this condition continues to impose a substantial disease burden (2). Owing to advances in surgical clipping, endovascular coiling, and neurocritical care protocols, the risk of rebleeding during the acute phase of aSAH and the perioperative management of this condition have become more consistently controlled. Nonetheless, secondary brain damage and its associated complications remain key determinants of patient prognosis (3, 4).

Delayed cerebral ischemia (DCI) constitutes a major secondary complication after aSAH and significantly influences both neurological recovery and overall clinical outcomes (5). DCI typically presents between several days and two weeks following the initial aSAH episode, manifesting as newly developed focal neurological deficits, reduced level of consciousness, or fresh ischemic lesions on neuroimaging. A multidisciplinary task force has proposed a standardized definition of DCI that integrates clinical neurological deterioration together with radiographically confirmed cerebral infarction within a comprehensive diagnostic framework (6). Moreover, contemporary reviews on DCI diagnosis and management emphasize that prompt recognition and treatment of DCI are essential components of post-aSAH neurocritical care (7). Consequently, early identification of patients at high risk for DCI upon hospital admission is essential for guiding monitoring intensity, performing risk stratification, and tailoring individualized treatment strategies.

The mechanisms underlying DCI are not confined to a single pathway. Historically, cerebral vasospasm was considered the primary contributor to DCI; however, clinical experience reveals that the severity of vasospasm does not always align with neurological deterioration. Current evidence suggests that multiple factors—including early brain injury after aSAH, local and systemic inflammatory responses, disruption of the blood–brain barrier, microcirculatory dysfunction, dysregulation of cerebral perfusion, and hemodynamic alterations—may collectively participate in DCI pathogenesis (8). Thus, depending solely on a single clinical grading scale or imaging parameter might inadequately capture DCI risk, whereas integrating information from diverse pathophysiological domains could more accurately reflect its multifactorial nature.

Macrophage migration inhibitory factor (MIF) is a multifunctional cytokine with immunomodulatory and pro-inflammatory properties, involved in innate immunity, inflammatory amplification, stress responses, and cellular signaling following tissue injury (9). Various cell types—including immune cells, endocrine cells, and endothelial cells—can secrete MIF, which then helps modulate systemic inflammation under conditions such as infection, trauma, surgical stress, and tissue damage (10). Furthermore, hypoxic stimulation induces MIF release from endothelial cells, indicating a relationship between MIF, ischemia, hypoxic stress, and endothelial reactivity (11). In the setting of aSAH, subarachnoid blood accumulation, its degradation products, and secondary inflammatory reactions may collectively trigger both local and systemic inflammation, thereby providing a rationale for considering MIF as an inflammation-associated biomarker of DCI risk after aSAH.

Although prior studies have investigated clinical predictors of DCI in aSAH patients, existing risk assessment methods have inherent limitations. For instance, several conventional tools primarily stratify risk using neurological scoring systems and the radiologic extent of hemorrhage, for example, by combining clinical grade with imaging findings to forecast DCI occurrence (12). In recent years, efforts have been directed toward constructing visual predictive models (e.g., nomograms) aimed at improving the interpretability and clinical applicability of personalized DCI risk evaluation (13). Nevertheless, current models predominantly emphasize clinical grades, imaging characteristics, or standard laboratory parameters, whereas the incremental benefit of incorporating MIF—an inflammation-related factor—with other clinical data remains underexplored. Hence, the question of how to combine inflammatory markers with routine clinical information to develop a simple, interpretable early risk prediction model merits further exploration.

Accordingly, the aim of this study was to examine the potential utility of admission serum MIF concentrations for DCI risk assessment following aSAH and to construct and internally validate an early risk assessment model centered on MIF. Through the integration of MIF and conventional clinical data, the discrimination, calibration, and clinical net benefit of the proposed model were evaluated, aiming to provide a straightforward and interpretable tool for early DCI risk stratification among patients with aSAH.

## Materials and methods

### Study population

This study included patients diagnosed with aSAH who were admitted to Nanjing Lishui People's Hospital between January 2021 and December 2025. All patients had subarachnoid hemorrhage confirmed by cranial computed tomography (CT), and the diagnosis of an intracranial aneurysm was further established using CT angiography, digital subtraction angiography, or relevant surgical findings. Patients were divided into a DCI group and a non-DCI group based on whether DCI occurred during hospitalization.

Inclusion criteria were as follows: (1) age ≥18 years; (2) meeting diagnostic criteria for aSAH; (3) completion of necessary clinical assessments, imaging examinations, and laboratory tests after admission; and (4) availability of clear data for determining DCI outcomes. Exclusion criteria were as follows: (1) traumatic subarachnoid hemorrhage, non-aSAH, or intracranial hemorrhage of other causes; (2) pre-existing severe post-stroke sequelae, intracranial tumors, or other central nervous system diseases that could significantly affect neurological evaluation; (3) concurrent severe infection, immune system diseases, malignant tumors, or other conditions that could markedly influence inflammatory marker levels; (4) missing key clinical data, imaging data, or MIF measurements; and (5) inability to assess DCI outcomes due to early death, discharge against medical advice, or incomplete data.

The study protocol was approved by the Ethics Committee of Nanjing Lishui People's Hospital (approval No. 2026KY0522-03). Given the retrospective nature of the study and the use of existing clinical data and archived residual serum samples, the requirement for written informed consent was waived by the Ethics Committee. All clinical data and serum samples were de-identified before analysis. The study was conducted in accordance with the Declaration of Helsinki and its subsequent amendments.

### Clinical data collection

Relevant patient data were collected from the hospital medical record system, imaging reports, and laboratory records. General information included age, sex, body mass index (BMI), smoking history, history of hypertension, and history of diabetes mellitus. Indicators of disease severity included the World Federation of Neurosurgical Societies (WFNS) grade at admission, modified Fisher (mFisher) grade, presence of intraventricular hemorrhage, and acute hydrocephalus. A high modified Fisher grade group was further defined as modified Fisher grades 3–4.

Aneurysm-related data included location, maximum diameter, and morphological characteristics. Aneurysm location was classified as anterior circulation or non-anterior circulation based on the site of the causative aneurysm. Aneurysm morphology was evaluated using imaging data, and the presence of irregular shape was recorded. Aneurysm-related imaging findings were comprehensively assessed based on imaging examinations and medical records.

Laboratory parameters included white blood cell (WBC) count, C-reactive protein (CRP), serum MIF, and high-sensitivity cardiac troponin I (hs-cTnI) measured early after admission. Hs-cTnI elevation was recorded according to the hospital's reference range. Cardiac function-related indicators included left ventricular ejection fraction (LVEF) obtained from echocardiography during hospitalization. Vascular ultrasound parameters included carotid artery resistance index and presence of moderate-to-severe carotid artery stenosis. Patient management, including aneurysm treatment and supportive or rescue measures when clinically indicated, was individualized by the treating team according to neurological status, aneurysm characteristics, imaging findings, and routine institutional practice.

### Serum MIF measurement

Archived residual serum samples obtained from peripheral venous blood collected during routine clinical care after admission were used for MIF measurement. Serum MIF levels were determined using enzyme-linked immunosorbent assay (ELISA) with a commercial MIF ELISA kit, following the manufacturer's instructions and standard laboratory quality-control protocols. MIF concentrations were expressed in ng/mL.

### Definition of DCI outcome

The primary outcome of this study was the occurrence of DCI during hospitalization. DCI was assessed throughout hospitalization by integrating serial neurological documentation, clinically indicated imaging findings, and the overall clinical course. DCI was defined as the appearance of a new focal neurological deficit or a decline in consciousness level after aSAH that could not be attributed to rebleeding, hydrocephalus, seizure, metabolic derangement, infection, sedative medications, or other identifiable causes; alternatively, DCI was defined by imaging evidence of new cerebral ischemia or infarction not explained by surgical or endovascular procedures or other definite etiologies. When direct neurological assessment was limited by sedation or poor neurological grade, the determination placed greater emphasis on changes from the documented neurological baseline, available imaging evidence, and exclusion of alternative causes. In ambiguous cases, the diagnosis was reviewed by neurosurgeons or neurointensivists based on the clinical course and imaging data.

### Statistical analysis

Statistical analyses were performed using R software. Continuous variables were first tested for normality. Normally distributed variables were expressed as mean ± standard deviation, and comparisons between groups were conducted using the independent samples *t*-test. Non-normally distributed variables were presented as median and interquartile range, with group comparisons performed using the Mann–Whitney U test. Categorical variables were expressed as counts and percentages, and group comparisons were made using the *χ*^2^ test or Fisher's exact test.

The occurrence of DCI was used as the dependent variable. Because serum MIF levels showed a skewed distribution, a natural logarithmic transformation was applied before inclusion in the models, followed by standardization. The effect of log(MIF) was interpreted per one-standard-deviation increase. For some continuous variables, appropriate scaling was performed to enhance the interpretability of the regression results based on clinical considerations. Categorical variables were coded according to clinical definitions or commonly used stratification criteria.

Univariable logistic regression analyses were performed to evaluate the association of each clinical variable with the risk of DCI. An expanded multivariable logistic regression model was used as an exploratory adjustment analysis incorporating clinically relevant variables and results of univariable analyses. Given the number of DCI events, the final risk assessment model was deliberately restricted to three clinically interpretable predictors to maintain parsimony and reduce the risk of overfitting. Predictor selection also considered model stability. A nomogram was developed based on the final multivariable logistic regression model to provide individualized prediction of DCI risk. Model results were presented as odds ratios (ORs) with 95% confidence intervals (CIs).

Receiver operating characteristic (ROC) curves were used to evaluate the discriminative ability of single indicators and the combined model for DCI, and the area under the curve (AUC), 95% CI, optimal cutoff value, sensitivity, and specificity were calculated. Comparisons of AUCs between different models were performed using the DeLong test. Calibration curves, the Brier score, calibration intercept, and calibration slope were used to assess the agreement between predicted probabilities and observed outcomes, and internal validation was conducted using bootstrap resampling. Decision curve analysis (DCA) was employed to evaluate the clinical net benefit of the model across different threshold probabilities. All tests were two-sided, and a *P* value <0.05 was considered statistically significant.

## Results

### Patients with DCI showed higher hemorrhage burden, elevated admission MIF levels, and reduced LVEF

A total of 100 patients with aSAH were included in this study, of whom 72 did not develop DCI and 28 developed DCI, yielding a DCI incidence of 28.0% (Figure 1). Baseline clinical characteristics of the two groups are summarized in Table 1.

**Figure 1:**
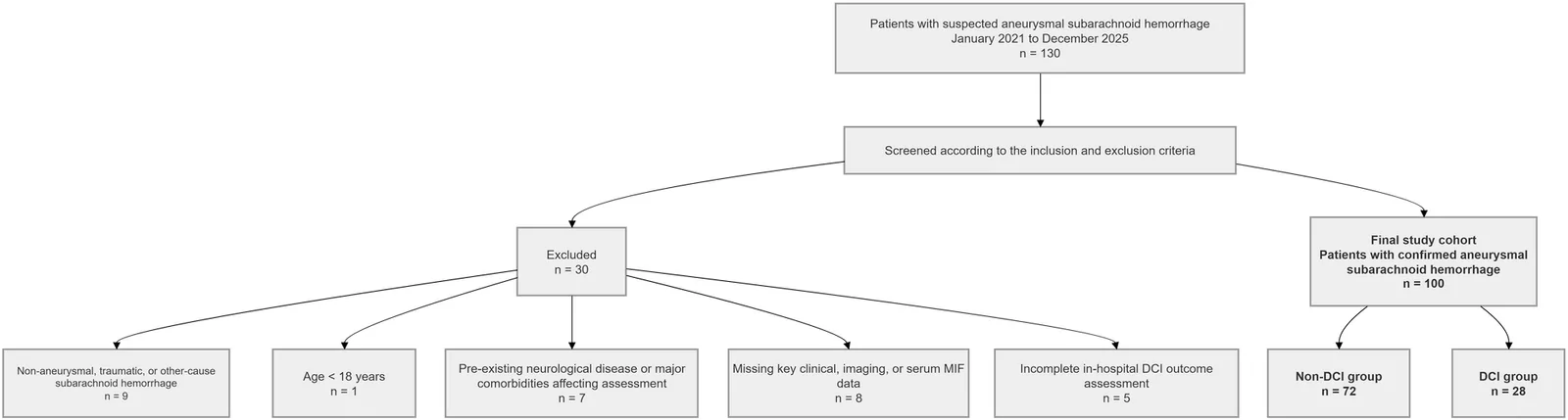
Flowchart of patient selection. Flowchart showing patient screening and inclusion in the final study cohort. Patients with suspected aneurysmal subarachnoid hemorrhage treated between January 2021 and December 2025 were screened according to the predefined inclusion and exclusion criteria. A total of 100 eligible patients with confirmed aneurysmal subarachnoid hemorrhage were included in the final analysis and classified into the DCI and non-DCI groups according to in-hospital delayed cerebral ischemia status. aSAH, aneurysmal subarachnoid hemorrhage; DCI, delayed cerebral ischemia; MIF, macrophage migration inhibitory factor.

**Table 1:** Baseline clinical, laboratory, imaging, and cardiac characteristics according to DCI status.

Variable	Total	Non-DCI	DCI	*P* value
Age, years	58.0 ± 10.9	56.8 ± 10.6	60.9 ± 11.4	0.113
BMI, kg/m^2^	24.3 ± 3.0	24.2 ± 3.0	24.2 (22.7–26.1)	0.392
Maximum aneurysm diameter, mm	6.6 (4.8–8.9)	6.3 (4.4–8.3)	7.6 (6.0–9.4)	0.054
Admission serum MIF, ng/mL	20.8 (17.0–25.2)	19.1 (16.3–23.1)	26.0 (19.9–32.5)	<0.001
WBC, 10⁹/L	11.5 (9.3–13.3)	11.3 ± 3.3	12.2 (10.5–14.7)	0.090
CRP, mg/L	8.4 (4.4–16.0)	7.0 (3.7–16.0)	11.1 (5.8–15.9)	0.048
LVEF, %	59.6 ± 7.0	61.0 ± 6.5	56.0 ± 7.0	0.002
Carotid resistance index	0.7 ± 0.1	0.7 ± 0.1	0.7 ± 0.1	0.091
Female	62 (62.0%)	43 (59.7%)	19 (67.9%)	0.601
Smoking history	29 (29.0%)	17 (23.6%)	12 (42.9%)	0.097
Hypertension	48 (48.0%)	31 (43.1%)	17 (60.7%)	0.173
Diabetes	12 (12.0%)	8 (11.1%)	4 (14.3%)	0.735
High WFNS grade	23 (23.0%)	13 (18.1%)	10 (35.7%)	0.105
Anterior circulation aneurysm	86 (86.0%)	60 (83.3%)	26 (92.9%)	0.338
Irregular aneurysm morphology	34 (34.0%)	21 (29.2%)	13 (46.4%)	0.161
mFisher				0.020
mFisher grade 1	12 (12.0%)	11 (15.3%)	1 (3.6%)	
mFisher grade 2	19 (19.0%)	17 (23.6%)	2 (7.1%)	
mFisher grade 3	30 (30.0%)	22 (30.6%)	8 (28.6%)	
mFisher grade 4	39 (39.0%)	22 (30.6%)	17 (60.7%)	
High mFisher grade	69 (69.0%)	44 (61.1%)	25 (89.3%)	0.013
Intraventricular hemorrhage	48 (48.0%)	30 (41.7%)	18 (64.3%)	0.070
Acute hydrocephalus	28 (28.0%)	16 (22.2%)	12 (42.9%)	0.069
Elevated hs-cTnI	28 (28.0%)	16 (22.2%)	12 (42.9%)	0.069
Moderate-to-severe carotid stenosis	11 (11.0%)	6 (8.3%)	5 (17.9%)	0.283

Data are presented as mean ± standard deviation, median (interquartile range), or *n* (%), as appropriate. Between-group comparisons were performed using the independent-samples *t*-test or Mann–Whitney U test for continuous variables and the chi-squared test or Fisher's exact test for categorical variables.

DCI, delayed cerebral ischemia; BMI, body mass index; MIF, macrophage migration inhibitory factor; WBC, white blood cell count; CRP, C-reactive protein; LVEF, left ventricular ejection fraction; WFNS, World Federation of Neurosurgical Societies; mFisher, modified Fisher; hs-cTnI, high-sensitivity cardiac troponin I.

Compared with patients without DCI, the DCI group showed a significantly different distribution of modified Fisher grade, with a higher proportion of patients with modified Fisher grade 4. Further analysis based on the high modified Fisher grade group revealed that the proportion of patients with modified Fisher grades 3–4 was significantly higher in the DCI group than in the non-DCI group. Admission serum MIF levels were markedly elevated in the DCI group (Figure 2), suggesting a more prominent inflammatory profile early after admission in patients who subsequently developed DCI. In addition, LVEF was lower in the DCI group than in the non-DCI group, indicating that cardiac function status may be associated with DCI risk.

**Figure 2:**
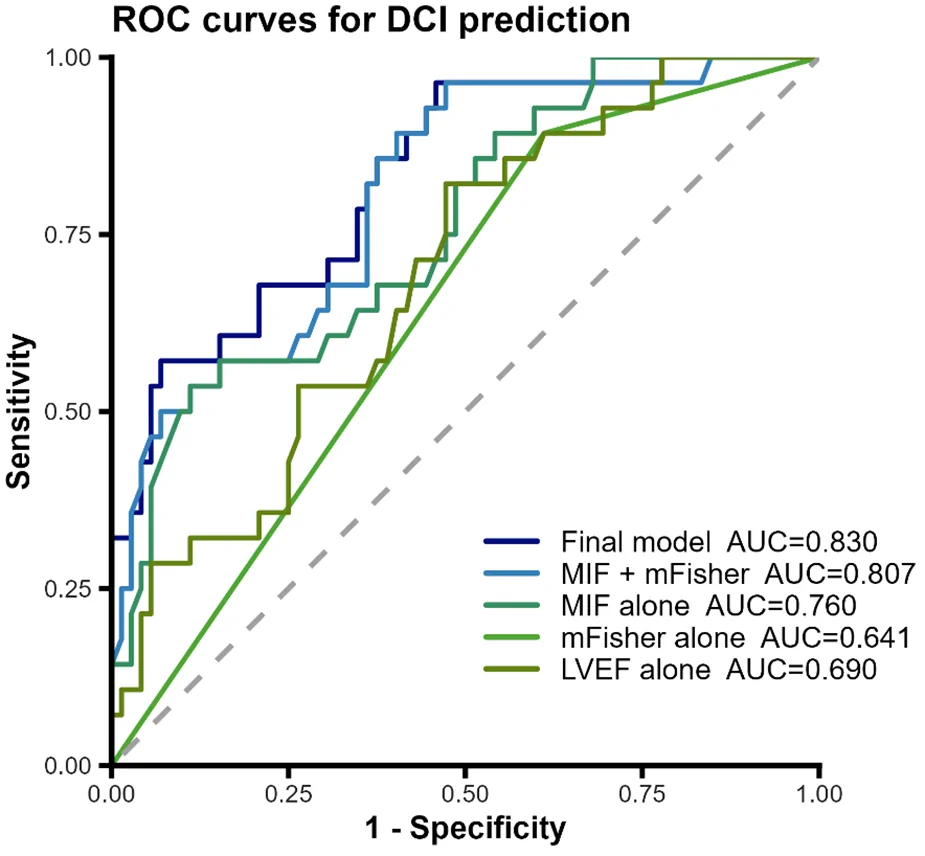
Serum MIF levels according to DCI status. Distribution of admission serum MIF levels in patients with and without DCI. Violin plots depict the overall distribution, boxplots indicate the median and interquartile range, and individual data points are shown. Between-group comparison was performed using the Wilcoxon rank-sum test. DCI, delayed cerebral ischemia; IQR, interquartile range; MIF, macrophage migration inhibitory factor.

Regarding inflammatory markers, CRP levels were higher in the DCI group than in the non-DCI group. WBC showed an increasing trend in the DCI group, but the difference did not reach statistical significance. Intraventricular hemorrhage, acute hydrocephalus, elevated high-sensitivity cardiac troponin I (hs-cTnI), and carotid artery resistance index were also more frequent or tended to be higher in the DCI group, but none of these differences reached statistical significance. No significant differences were observed between the two groups in age, sex, BMI, smoking history, hypertension, diabetes mellitus, high WFNS grade group, aneurysm location, maximum aneurysm diameter, irregular aneurysm morphology, or moderate-to-severe carotid artery stenosis.

### Elevated admission MIF, high modified fisher grade, and reduced LVEF were associated with increased DCI risk

Results of univariable logistic regression analyses are presented in Table 2. Elevated admission MIF level was significantly associated with an increased risk of DCI; each one-standard-deviation increase in log(MIF) was associated with an approximately 3.27-fold higher risk of DCI. High modified Fisher grade was also associated with DCI occurrence, with patients with modified Fisher grades 3–4 having a higher risk of DCI than those in the low modified Fisher grade group. Reduced LVEF was likewise associated with an increased risk of DCI; each 5% decrease in LVEF was associated with an increased DCI risk.

**Table 2:** Univariable logistic regression analysis of factors associated with DCI.

Variable	OR (95% CI)	*P* value
Age, years	1.04 (0.99–1.08)	0.101
Female	1.42 (0.57–3.58)	0.453
BMI, kg/m^2^	1.07 (0.92–1.24)	0.396
Smoking history	2.43 (0.96–6.12)	0.060
Hypertension	2.04 (0.84–4.98)	0.116
Diabetes	1.33 (0.37–4.84)	0.662
High WFNS grade	2.52 (0.95–6.71)	0.064
Anterior circulation aneurysm	2.60 (0.54–12.45)	0.232
Maximum aneurysm diameter, mm	1.08 (0.97–1.20)	0.168
Irregular aneurysm morphology	2.10 (0.86–5.18)	0.105
High mFisher grade	5.30 (1.46–19.22)	0.011
Intraventricular hemorrhage	2.52 (1.02–6.22)	0.045
Acute hydrocephalus	2.62 (1.03–6.67)	0.042
log(MIF), per 1-SD increase	3.27 (1.80–5.96)	<0.001
WBC, per 1-SD increase	1.60 (1.03–2.48)	0.037
CRP, per 1-SD increase	1.24 (0.81–1.89)	0.318
LVEF, per 5% decrease	1.74 (1.22–2.47)	0.002
Elevated hs-cTnI	2.62 (1.03–6.67)	0.042
Moderate-to-severe carotid stenosis	2.39 (0.67–8.59)	0.181

Odds ratios were calculated using univariable logistic regression models. Continuous variables were analyzed per unit increase unless otherwise specified. log(MIF) was analyzed per 1-standard deviation increase after natural logarithmic transformation and standardization. WBC and CRP were analyzed per 1-standard deviation increase. LVEF was analyzed per 5% decrease. High mFisher grade was defined as mFisher grade 3–4.

DCI, delayed cerebral ischemia; OR, odds ratio; CI, confidence interval; BMI, body mass index; WFNS, World Federation of Neurosurgical Societies; MIF, macrophage migration inhibitory factor; WBC, white blood cell count; CRP, C-reactive protein; LVEF, left ventricular ejection fraction; hs-cTnI, high-sensitivity cardiac troponin I; mFisher, modified Fisher.

In addition to the above major variables, intraventricular hemorrhage, acute hydrocephalus, elevated WBC count, and elevated hs-cTnI were also associated with an increased risk of DCI. High WFNS grade showed a borderline association with DCI occurrence but did not reach statistical significance. Although CRP differed between groups at baseline, it did not show a significant association in univariable logistic regression.

### MIF, high modified fisher grade, and LVEF remained independently associated with DCI in candidate multivariable models

To further evaluate the independent association of admission MIF with DCI occurrence, candidate multivariable logistic regression models were constructed incorporating MIF, high modified Fisher grade, LVEF, and several clinically relevant variables. The results are shown in Table 3. After adjustment for high WFNS grade, intraventricular hemorrhage, acute hydrocephalus, WBC count, and CRP, each one-standard-deviation increase in log(MIF) remained significantly associated with an increased risk of DCI (adjusted OR = 3.51, 95% CI: 1.67–7.36, *P* < 0.001). High modified Fisher grade was also independently associated with DCI occurrence (adjusted OR = 8.78, 95% CI: 1.28–60.26, *P* = 0.027). Each 5% decrease in LVEF remained associated with an increased risk of DCI (adjusted OR = 1.54, 95% CI: 1.02–2.33, *P* = 0.042).

**Table 3:** Candidate multivariable logistic regression model for DCI.

Variable	OR (95% CI)	*P* value
log(MIF), per 1-SD increase	3.51 (1.67–7.36)	<0.001
High mFisher grade	8.78 (1.28–60.26)	0.027
LVEF, per 5% decrease	1.54 (1.02–2.33)	0.042
High WFNS grade	1.75 (0.40–7.69)	0.456
Intraventricular hemorrhage	0.48 (0.10–2.44)	0.380
Acute hydrocephalus	1.30 (0.24–7.23)	0.761
WBC, per 1-SD increase	0.83 (0.44–1.55)	0.556
CRP, per 1-SD increase	0.85 (0.48–1.48)	0.556

The candidate multivariable model included variables selected according to clinical relevance, univariable analysis results, and model stability. log(MIF) was analyzed per 1-standard deviation increase after natural logarithmic transformation and standardization. WBC and CRP were analyzed per 1-standard deviation increase. LVEF was analyzed per 5% decrease. High mFisher grade was defined as mFisher grade 3–4.

DCI, delayed cerebral ischemia; OR, odds ratio; CI, confidence interval; MIF, macrophage migration inhibitory factor; LVEF, left ventricular ejection fraction; WFNS, World Federation of Neurosurgical Societies; WBC, white blood cell count; CRP, C-reactive protein; mFisher, modified Fisher.

In contrast, high WFNS grade, intraventricular hemorrhage, acute hydrocephalus, WBC count, and CRP did not retain statistical significance in the candidate multivariable models. Based on statistical performance, clinical interpretability, and model stability, the final model incorporated three variables: log(MIF), high modified Fisher grade, and LVEF.

### The final three-variable model showed that MIF, hemorrhage burden, and cardiac function were each independently associated with DCI risk

Results of the final logistic regression model are presented in Table 4. Each one-standard-deviation increase in log(MIF) was significantly associated with an increased risk of DCI (adjusted OR = 3.06, 95% CI: 1.61–5.79, *P* < 0.001). Patients with modified Fisher grades 3–4 had a higher risk of DCI than those in the low modified Fisher grade group (adjusted OR = 6.20, 95% CI: 1.25–30.74, *P* = 0.025). Furthermore, each 5% decrease in LVEF was associated with an increased risk of DCI (adjusted OR = 1.49, 95% CI: 1.01–2.19, *P* = 0.044).

**Table 4:** Final multivariable logistic regression model for DCI risk assessment.

Variable	OR (95% CI)	*P* value
log(MIF), per 1-SD increase	3.06 (1.61–5.79)	<0.001
High mFisher grade	6.20 (1.25–30.74)	0.025
LVEF, per 5% decrease	1.49 (1.01–2.19)	0.044

The final model included log(MIF), high mFisher grade, and LVEF. log(MIF) was analyzed per 1-standard deviation increase after natural logarithmic transformation and standardization. LVEF was analyzed per 5% decrease. High mFisher grade was defined as mFisher grade 3–4.

DCI, delayed cerebral ischemia; OR, odds ratio; CI, confidence interval; MIF, macrophage migration inhibitory factor; mFisher, modified Fisher; LVEF, left ventricular ejection fraction.

These findings indicate that admission MIF level, radiographic hemorrhage burden, and cardiac function status are each independently associated with the risk of DCI in patients with aSAH and together constitute the core variables of the subsequent risk assessment model.

### The MIF-based combined model showed good discriminative performance for DCI

Model discrimination performance is presented in Table 5 and Figure 3. Using MIF alone to predict DCI yielded an AUC of 0.760 (95% CI: 0.657–0.864), with an optimal cutoff value of 25.6 ng/mL, corresponding to a sensitivity of 0.536 and a specificity of 0.889. High modified Fisher grade alone predicted DCI with an AUC of 0.641 (95% CI: 0.560–0.722), a sensitivity of 0.893, and a specificity of 0.389. LVEF alone predicted DCI with an AUC of 0.690 (95% CI: 0.580–0.801), a sensitivity of 0.821, and a specificity of 0.528.

**Table 5:** Discriminative performance of individual predictors and combined models for DCI prediction.

Model	AUC (95% CI)	Cutoff	Sensitivity	Specificity
MIF alone	0.760 (0.657–0.864)	25.6	0.536	0.889
mFisher alone	0.641 (0.560–0.722)	0.5	0.893	0.389
LVEF alone	0.690 (0.580–0.801)	≤61.6%	0.821	0.528
MIF+mFisher	0.807 (0.714–0.900)	0.155	0.964	0.528
Final model	0.830 (0.743–0.918)	0.152	0.964	0.542

The discriminative performance of each predictor and model was evaluated using ROC curve analysis. Cutoff values were determined using the Youden index. For the MIF+mFisher model and the final model, the cutoff represents the predicted probability. For high mFisher grade, the cutoff of 0.5 corresponds to classification as the high mFisher group. For LVEF, the cutoff is presented on the original percentage scale after back-transformation from the model coding, and lower LVEF values indicate higher predicted DCI risk.

AUC, area under the receiver operating characteristic curve; CI, confidence interval; DCI, delayed cerebral ischemia; MIF, macrophage migration inhibitory factor; mFisher, modified Fisher; LVEF, left ventricular ejection fraction.

**Figure 3:**
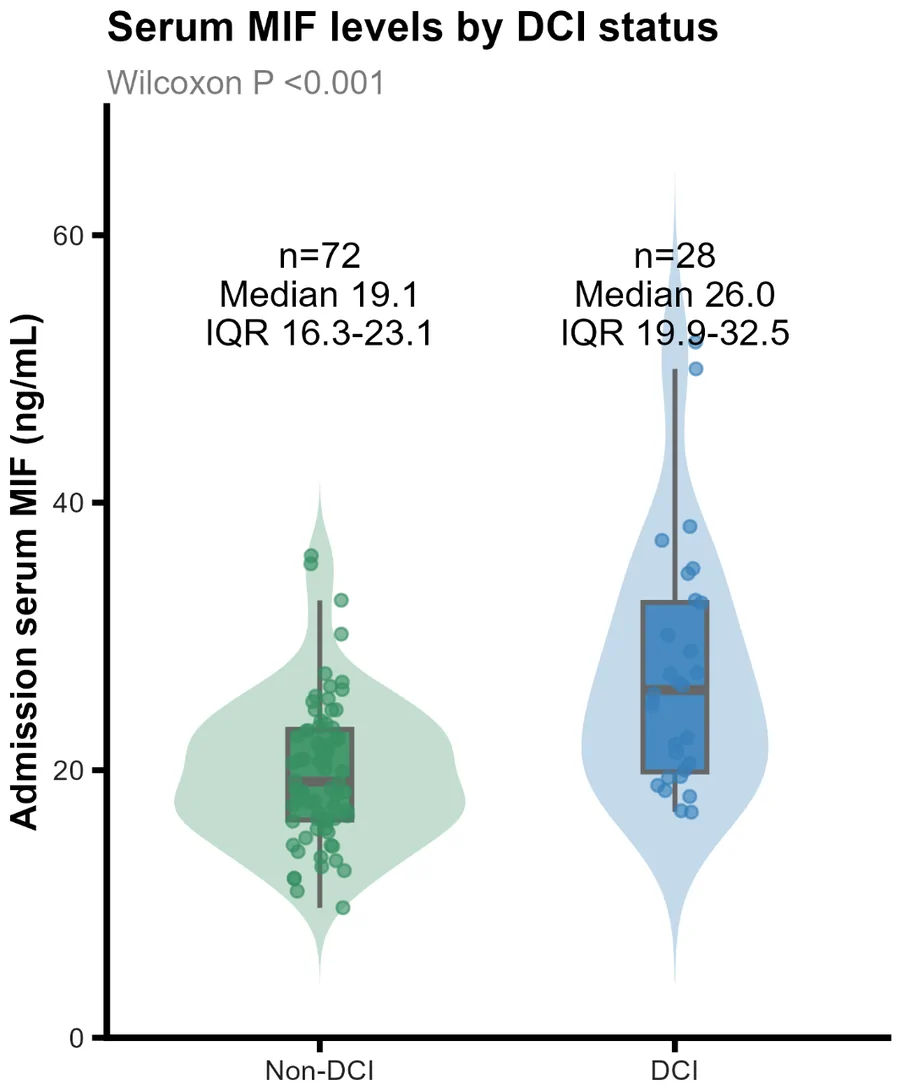
Receiver operating characteristic curves for DCI prediction. Receiver operating characteristic curves comparing the discriminative performance of the final model and individual predictors for delayed cerebral ischemia after aneurysmal subarachnoid hemorrhage. The final model incorporated serum MIF, high modified Fisher grade, and LVEF. AUC values are shown in the figure. ROC, receiver operating characteristic; AUC, area under the curve; DCI, delayed cerebral ischemia; LVEF, left ventricular ejection fraction; MIF, macrophage migration inhibitory factor.

When MIF was combined with high modified Fisher grade, the AUC increased to 0.807 (95% CI: 0.714–0.900), with a sensitivity of 0.964 and a specificity of 0.528. After further incorporating LVEF, the final model achieved an AUC of 0.830 (95% CI: 0.743–0.918), with a sensitivity of 0.964 and a specificity of 0.542, representing the highest overall discriminative ability.

DeLong tests showed that the AUC of the final model was significantly higher than that of MIF alone (*P* = 0.013), high modified Fisher grade alone (*P* < 0.001), and LVEF alone (*P* = 0.007). Compared with the base model combining MIF and high modified Fisher grade, the final model showed a further improvement in AUC, although the difference did not reach statistical significance (*P* = 0.218). Moreover, the AUC of the model combining MIF and high modified Fisher grade was significantly higher than that of MIF alone (*P* = 0.035), suggesting that integrating hemorrhage burden information with MIF further improved DCI discrimination.

### Construction of an individualized risk prediction nomogram for DCI based on the final model

Based on the final multivariable logistic regression model, a nomogram was constructed incorporating log(MIF), high modified Fisher grade, and LVEF to estimate the individualized risk of DCI in patients with aSAH (Figure 4).

**Figure 4:**
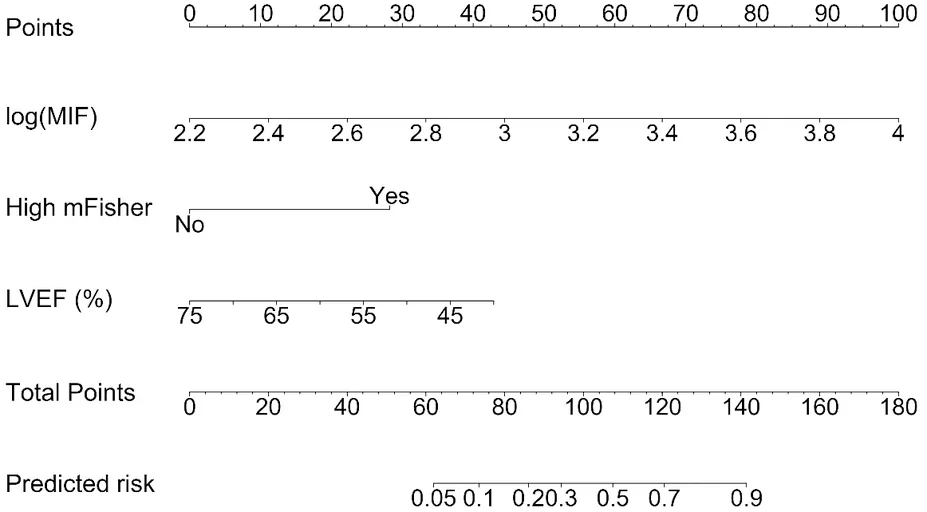
Nomogram for individualized prediction of DCI risk. Nomogram constructed from the final multivariable logistic regression model to estimate the individualized risk of delayed cerebral ischemia after aneurysmal subarachnoid hemorrhage. Points are assigned according to log-transformed serum MIF, high modified Fisher grade, and LVEF, and the total points correspond to the predicted probability of DCI. DCI, delayed cerebral ischemia; LVEF, left ventricular ejection fraction; MIF, macrophage migration inhibitory factor.

Regarding the contribution of each variable to the nomogram score, log(MIF) contributed the most, with a relative score of 100.0, followed by reduced LVEF with a relative score of 42.0 and high modified Fisher grade with a relative score of 30.2. The relationship between total score and predicted risk was non-linear and increasing. As the total score increased, the predicted risk of DCI rose markedly. Based on model estimates, a total score of approximately 75 corresponded to a predicted DCI risk of about 11.3%, a score of approximately 100 corresponded to a risk of 36.5%, a score of around 125 corresponded to a risk of 72.2%, and a score of about 150 corresponded to a risk of 92.2%. These results indicate that the nomogram integrates MIF level, hemorrhage burden, and cardiac function status into a straightforward tool for individualized risk estimation.

### Calibration and internal validation of the final model

Calibration analysis of the final model is shown in Figure 5A. The Brier score of the model was 0.140, indicating good overall agreement between predicted probabilities and observed outcomes. The apparent calibration intercept was close to 0, and the calibration slope was 1.000, suggesting a good fit of the model to the original data.

**Figure 5:**
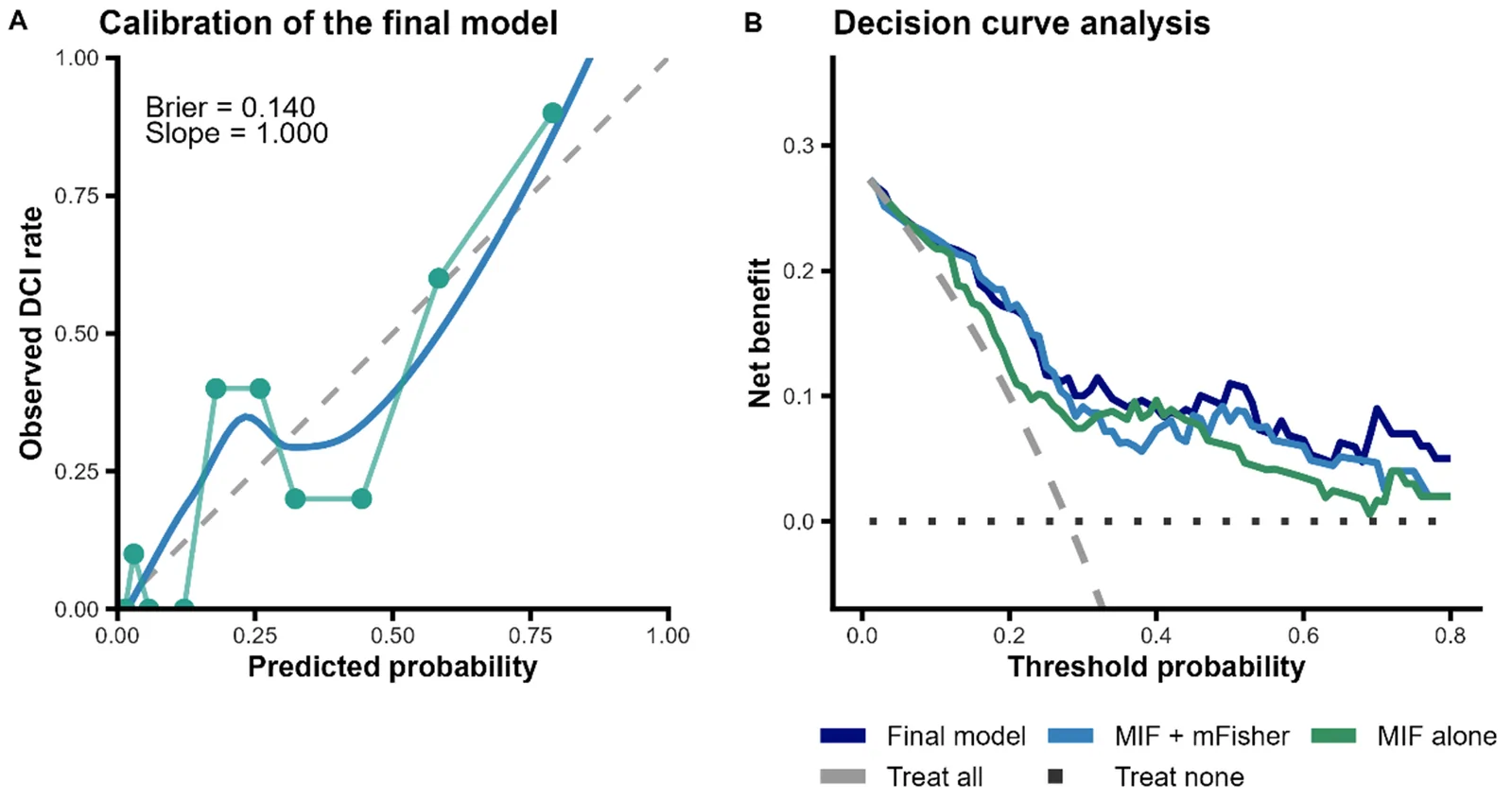
Calibration and decision curve analysis of the final model. Model performance evaluation of the final DCI risk assessment model. Panel **A** displays the calibration plot comparing predicted probabilities with observed DCI rates. The dashed diagonal line represents perfect calibration. Panel **B** presents the decision curve analysis evaluating the clinical net benefit of the final model across different threshold probabilities, compared with the MIF + mFisher model, MIF alone, the treat-all strategy, and the treat-none strategy. DCA, decision curve analysis; DCI, delayed cerebral ischemia; MIF, macrophage migration inhibitory factor.

Internal validation was performed using 500 bootstrap resamples. The apparent AUC of the final model was 0.830, and the optimism-corrected AUC was 0.818, indicating that the model retained good discriminative ability after internal validation. The corrected Brier score was 0.153, and the corrected calibration slope was 0.902, suggesting some degree of optimism in the model, but overall calibration remained stable. Combined with the calibration curve, the predicted risks of the final model were generally consistent with observed risks within the derivation cohort, supporting further external evaluation of the model.

### DCA demonstrated potential clinical net benefit of the final model

Results of the DCA are presented in Figure 5B. Compared with the two reference strategies (“treat all as high risk” and “treat none”), the final model showed a higher net benefit across a threshold probability range of 0.05–0.80, indicating potential applicability under common clinical decision thresholds. When compared with single predictors and the base model combining MIF with high modified Fisher grade, the decision curve of the final model exhibited overall superior or comparable net benefit, suggesting that further incorporating LVEF along with MIF and hemorrhage burden provides additional clinical decision-making information for DCI risk assessment. In summary, the final model demonstrated favorable internal performance in terms of discrimination, calibration, and potential clinical net benefit.

## Discussion

The present study focused on early risk assessment of DCI following aSAH. Patients who experienced DCI exhibited greater radiographic hemorrhage burden, higher admission serum MIF concentrations, and lower LVEF values. Multivariable analyses further demonstrated that log(MIF), a high modified Fisher grade, and reduced LVEF each retained independent associations with DCI risk. A preliminary model combining these three variables showed favorable discrimination, calibration, and potential clinical utility within the derivation cohort. These observations imply that post-aSAH DCI risk evaluation should move beyond any single clinical or imaging indicator and instead incorporate multiple dimensions such as inflammatory activity, initial hemorrhage burden, and cardiac function.

DCI represents a frequent and serious complication that strongly influences patient outcomes after aSAH, and its pathophysiology remains multifactorial. According to published literature, DCI develops in roughly 30% of aSAH cases and constitutes a major reason for poor neurological recovery among survivors (14). Earlier concepts primarily linked DCI to large-artery vasospasm; however, accumulating evidence suggests that vasospasm alone cannot fully account for DCI occurrence (15–17). Beyond large-vessel narrowing, other mechanisms—including early brain injury, neuroinflammation, microcirculatory impairment, microthrombus formation, disruption of the blood–brain barrier, cortical spreading depolarization, failure of cerebral autoregulation, and responses to blood breakdown products—may all contribute to DCI pathogenesis (18, 19). Consequently, using a sole imaging score or a single laboratory parameter might be insufficient to capture DCI risk. The final model developed in this work integrated MIF, high modified Fisher grade, and LVEF, each reflecting distinct pathophysiological domains (inflammation, hemorrhage burden, and cardiac function), thereby enabling a more comprehensive risk assessment.

In this cohort, admission serum MIF levels were markedly higher in the DCI group and remained independently associated with DCI across candidate multivariable models as well as the final model. MIF is a cytokine possessing both immunomodulatory and pro-inflammatory activities; it is expressed by diverse cell types including immune cells, endocrine cells, epithelial cells, endothelial cells, neurons, and glial cells. Under pathological conditions such as hypoxia, infection, surgical stress, or tissue injury, MIF can be released and can participate in orchestrating local and systemic inflammatory reactions (20). After aSAH, extravasated blood within the subarachnoid space triggers inflammatory cell activation, blood–brain barrier compromise, and alterations in the cerebral microenvironment, and inflammation has been recognized as a key contributor to DCI susceptibility (20). Prior clinical studies have reported that serum MIF levels in aSAH patients correlate with WFNS grade, Fisher grade, inflammatory markers, and brain injury biomarkers, and can predict unfavorable 6-month functional outcomes (21). Additional work demonstrated that admission serum MIF levels are significantly elevated in aSAH patients who later develop DCI, and that adding MIF to established risk factors improves both discrimination and reclassification for DCI prediction (22). Moreover, elevated MIF in cerebrospinal fluid has been associated with cerebral vasospasm and clinical prognosis (23). Neumaier and colleagues documented temporal MIF changes across serum, cerebrospinal fluid, and cerebral microdialysate after aSAH, noting higher MIF levels in serum and local microdialysate of DCI patients (20). The current findings are consistent with this body of evidence, indicating that high admission MIF may reflect the intensity of acute inflammatory activation and the likelihood of secondary brain injury after aSAH. MIF not only demonstrated reasonable discriminative power as a standalone marker but also contributed a substantial relative score in the nomogram, highlighting its potential as a heavily weighted inflammation-related biomarker in DCI risk assessment.

High modified Fisher grade also emerged as an independent predictor of DCI in this study. The modified Fisher grade evaluates subarachnoid clot thickness and the presence of intraventricular hemorrhage; it is a widely used radiographic tool for estimating initial hemorrhage burden and predicting subsequent cerebrovascular events after aSAH. Previous investigations have shown that both subarachnoid blood and intraventricular hemorrhage increase DCI risk, possibly with an additive effect (24). Compared with the original Fisher scale, the modified version incorporates intraventricular blood information and better captures hemorrhage characteristics linked to symptomatic vasospasm (25). Systematic reviews have confirmed that the extent of subarachnoid hemorrhage is a strong DCI predictor, and scales such as the Fisher grade, mFisher grade, and clot volume scores are frequently employed in DCI risk stratification (26). Mechanistically, a larger hemorrhage burden may promote DCI via multiple pathways, including stimulation by blood degradation products, oxidative stress, endothelial injury, amplified inflammation, impaired cerebrospinal fluid circulation, and microcirculatory damage. Quantitative assessments of blood volume also support an association between clot burden and DCI risk (27). Among different compartments, cisternal blood volume may correlate more closely with DCI than blood in other locations (28). In the present study, modified Fisher grades 3–4 were designated as the high-grade group and included in the final model. After adjustment for MIF and LVEF, high modified Fisher grade remained significantly associated with increased DCI risk, confirming that traditional imaging-derived hemorrhage burden remains a valuable component of DCI risk evaluation. Notably, the discriminative ability of high modified Fisher grade alone was modest, but its combination with MIF yielded a higher AUC, suggesting complementarity between radiographic hemorrhage burden and inflammatory markers.

Reduced LVEF was another independent factor retained in the final model. Following aSAH, dysfunction of the brain-heart axis and neurogenic myocardial injury can occur. The underlying mechanisms involve sympathetic overactivation, excessive catecholamine release, and abnormal cardiac sympathetic innervation, which clinically manifest as elevated cardiac enzymes, electrocardiographic anomalies, regional wall motion abnormalities, and decreased left ventricular systolic function (29, 30). Prior studies have indicated that left ventricular segmental wall motion abnormalities after aSAH are associated with disease severity, troponin elevation, and heart rate changes (31). Cardiac dysfunction not only reflects the degree of cardiac involvement but may also influence DCI or ischemic injury by affecting systemic hemodynamic stability, cerebral perfusion reserve, and the capacity to compensate for secondary ischemia. Existing evidence suggests that left ventricular dysfunction increases the risk of vasospasm-related cerebral infarction after aSAH and is linked to adverse factors such as hypotension and pulmonary edema (32). A meta-analysis further concluded that cardiac injury and dysfunction after aSAH are associated with elevated risks of death, poor outcome, and DCI (33). In addition, prospective multicenter data have shown that cardiac abnormalities after aSAH correlate with DCI and clinical outcomes, and this relationship persists after adjusting for certain conventional risk factors (34). The results of the current study suggest that LVEF provides incremental information beyond standard neurological and imaging indicators, underscoring the importance of considering brain-heart interactions and circulatory function in DCI risk assessment.

The final model developed here possesses good clinical interpretability. By incorporating MIF (inflammation), high modified Fisher grade (hemorrhage burden), and LVEF (cardiac status), the model captures DCI risk from complementary pathophysiological angles. Compared with single predictors, the combined model integrates admission serum biomarkers, a conventional imaging grade, and an echocardiographic parameter into a relatively simple yet clinically meaningful framework. Regarding model performance, the final model achieved a higher AUC than any individual predictor, and DeLong tests indicated significant improvements over MIF alone, high modified Fisher grade alone, and LVEF alone. When compared with the base model (MIF plus high modified Fisher grade), adding LVEF further increased the AUC; although this increase did not reach statistical significance, it suggests that cardiac functional status may offer supplementary information beyond inflammation and bleeding severity for DCI risk prediction.

To improve clinical usability, a nomogram was constructed based on the final three-variable model, converting MIF, high modified Fisher grade, and LVEF into a visual tool for individualized risk estimation. In practical application, clinicians can assign points to each variable according to the patient's admission serum MIF level, whether the modified Fisher grade is high, and the measured LVEF. Summing these points yields a total score, and the corresponding predicted probabilit*y* axis provides an individualized DCI risk estimate. Thus, model outputs are no longer limited to overall AUC or OR values but can be directly translated into patient-specific probabilities. Calibration curves and bootstrap internal validation showed acceptable agreement between predicted and observed risks within the derivation cohort; however, these analyses do not establish external generalizability.

From a clinical standpoint, DCA further illustrated the model's potential value in risk stratification. In essence, DCA assesses whether using a model to guide decisions—such as classifying a patient as high-risk for DCI and thereby instituting closer monitoring or more aggressive management—offers a higher net clinical benefit compared with two extreme strategies: treating all patients as high risk or treating none as high risk. The present results showed that the final model provided a higher net benefit than these extreme strategies across several clinically relevant threshold probability ranges, and outperformed single predictors and the base model (MIF plus high modified Fisher grade) within certain thresholds. In other words, the model may help reduce unnecessary intensive management in low-risk individuals while minimizing the likelihood of overlooking high-risk patients.

It should be stressed that this model is not meant to replace clinical judgment, nor does it imply that all patients with a high predicted risk should receive uniform interventions. Management of DCI still requires integrated consideration of the patient's level of consciousness, neurological signs, imaging findings, evidence of vasospasm, cerebral perfusion status, and center-specific protocols. In this retrospective cohort, management strategies were individualized according to clinical indications, and residual confounding related to treatment differences cannot be completely excluded. The primary utility of this model lies in assisting clinicians to systematically organize early-admission information and identify individuals who warrant closer observation and serial assessments. Overall, MIF offers a novel perspective on inflammation-related risk evaluation after aSAH, high modified Fisher grade retains traditional imaging-based hemorrhage burden information, and LVEF adds the dimension of brain-heart interaction and circulatory reserve. Combined, these three variables provide a relatively intuitive adjunctive reference for early DCI risk stratification in patients with aSAH without substantially increasing the burden of data collection.

Several limitations of this work should be acknowledged. First, this was a single-center retrospective study, and institution-specific patient characteristics, management practices, and laboratory procedures may limit the generalizability of the findings. The cohort included 100 patients and 28 DCI events; although the final model was kept parsimonious and internally validated using bootstrap resampling, effect estimates may remain imprecise and susceptible to overfitting. Accordingly, the model should be considered preliminary and exploratory until externally validated. Second, the study primarily used early-admission MIF levels, and dynamic changes in MIF were not examined. Future prospective studies with standardized blood-sampling time points and serial MIF measurements may clarify the temporal relationship between MIF changes and DCI onset. Third, future studies incorporating standardized assessment of cerebral vasospasm, detailed treatment documentation, and longer-term functional outcome evaluation may further clarify the mechanistic and prognostic significance of MIF. Finally, bootstrap validation cannot replace independent external validation; larger multicenter prospective cohorts are needed to confirm model performance and clinical applicability.

In summary, this study found that elevated admission serum MIF level, high modified Fisher grade, and reduced LVEF were each independently associated with an increased risk of DCI in patients with aSAH. The preliminary risk assessment model based on these three indicators demonstrated favorable internal discrimination, calibration, and potential clinical net benefit, thereby supporting its further evaluation as a simple and interpretable tool for early DCI risk stratification.

## Conclusions

Elevated admission serum MIF concentration, a high modified Fisher grade, and decreased LVEF were each independently associated with an increased risk of DCI in patients with aSAH. An internally validated risk assessment model incorporating these three indicators demonstrated favorable discrimination, calibration, and potential clinical net benefit within the derivation cohort. This model integrates three domains—inflammatory response, hemorrhage burden, and cardiac functional status—and may serve as a simple, interpretable adjunctive tool for early DCI risk stratification. Its generalizability and clinical applicability require confirmation in larger, multicenter prospective cohorts.

## Data Availability

The raw data supporting the conclusions of this article will be made available by the authors, without undue reservation.
